# Roles of Lactose and Fructose Malabsorption and Dietary Outcomes in Children Presenting with Chronic Abdominal Pain

**DOI:** 10.3390/nu11123063

**Published:** 2019-12-16

**Authors:** Carsten Posovszky, Vreni Roesler, Sebastian Becker, Enno Iven, Christian Hudert, Friedrich Ebinger, Claudia Calvano, Petra Warschburger

**Affiliations:** 1 University Medical Centre Ulm, Department for Pediatric and Adolescent Medicine, Pediatric Gastroenterology, 89075 Ulm, Germany; vreni.roesler@uniklinik-ulm.de; 2 Darmstädter Kinderkliniken Prinzessin Margaret, Dieburger Str. 31, 64287 Darmstadt, Germany; Sebastian.Becker@alice-hospital.de; 3 Katholisches Kinderkrankenhaus Wilhelmsstift, Liliencronstr.130, 22149 Hamburg, Germany; e.iven@kkh-wilhelmstift.de; 4 Charité–Universitätsmedizin Berlin, corporate member of Freie Universität Berlin, Humboldt-Universität zu Berlin and Berlin Institute of Health, Department of Pediatric Gastroenterology, Augustenburger Platz 1, 13353 Berlin, Germany; christian.hudert@charite.de; 5 St. Vincenz-Krankenhaus GmbH Paderborn, Klinik für Kinder- und Jugendmedizin, Husener Str. 81, 33098 Paderborn, Germany; F.Ebinger@vincenz.de; 6 Charité–Universitätsmedizin Berlin, corporate member of Freie Universität Berlin, Humboldt-Universität zu Berlin and Berlin Institute of Health, Department of Child and Adolescent Psychiatry, Augustenburger Platz 1, 13353 Berlin, Germany; 7 University of Potsdam, Department Psychology, Counseling Psychology, Karl-Liebknecht-Str. 24-25, 14476 Potsdam, Germany; warschb@uni-potsdam.de

**Keywords:** chronic abdominal pain, children, fructose malabsorption, lactose intolerance, hydrogen breath test, functional abdominal pain disorders

## Abstract

Intolerance to lactose or fructose is frequently diagnosed in children with chronic abdominal pain (CAP). However, the causal relationship remains a matter of discussion. A cohort of 253 patients, aged 7–12 years, presenting with unexplained CAP received standardized diagnostics. Additional diagnostic tests were performed based on their medical history and physical and laboratory investigations. Fructose and lactose hydrogen breath tests (H_2_BT) as well as empiric diagnostic elimination diets were performed in 135 patients reporting abdominal pain related to the consumption of lactose or fructose to evaluate carbohydrate intolerance as a potential cause of CAP. Carbohydrate malabsorption by H2BT was found in 55 (41%) out of 135 patients. An abnormal increase in H2BT was revealed in 30% (35/118) of patients after fructose consumption and in 18% (20/114) of patients after lactose administration. Forty-six percent (25/54) reported pain relief during a diagnostic elimination diet. In total, 17 patients had lactose malabsorption, 29 fructose malabsorption, and nine combined carbohydrate malabsorption. Carbohydrate intolerance as a cause of CAP was diagnosed at follow-up in only 18% (10/55) of patients with malabsorption after the elimination of the respective carbohydrate. Thus, carbohydrate malabsorption appears to be an incidental finding in children with functional abdominal pain disorders, rather than its cause. Therefore, testing of carbohydrate intolerance should only be considered in children with a strong clinical suspicion and with the goal to prevent long-term unnecessary dietary restrictions in children suffering from CAP.

## 1. Introduction

Chronic abdominal pain (CAP) is one of the most frequent complaints in children and adolescents with prevalence rates ranging from 0.3% to 19% in Western countries [1,2]. CAP is referred to as functional abdominal pain disorder (FAPD), according to the pediatric ROME IV classification, if it cannot be attributed to another medical condition after appropriate medical evaluation [3,4]. CAP is mostly considered functional; however, in up to 45% of cases, organic abnormalities are found [5,6]. Lactose and fructose malabsorption are frequently reported in children suffering from CAP [7,8,9,10,11]. 

Food intolerance is defined as a non-immunological response initiated by a food or food component at a normally tolerated dose and accounts for most adverse food reactions [12,13]. Abdominal pain, diarrhea, flatulence, and nausea are common symptoms that occur after the ingestion of juice and dairy products due to osmotic effects and fermentation processes of the colonic microbiota in carbohydrate intolerant children [14]. 

Primary lactase deficiency or hypolactasia is the most common cause of lactose malabsorption and intolerance worldwide. Hypolactasia typically develops between two and 20 years of age due to hereditary reduced lactase expression and activity [14,15]. Secondary lactose malabsorption results from impaired lactase activity due to bacterial overgrowth of the small intestine, infectious enteritis, celiac disease, drug use, or inflammatory bowel disease [14]. In addition, intestinal fructose intolerance is a growing cause of CAP in children, as fructose is increasingly being consumed by children due to its widespread usage as a sweetener [14,16]. 

In light of the high prevalence of carbohydrate malabsorption in children presenting with CAP [7,8,9,10,11], diagnostics should include the investigation of lactose or fructose intolerance if a diet-related cause is assumed. We investigated children referred with CAP within the framework of a randomized controlled trial for the evaluation of a cognitive behavioral training program for FAPD (NCT02030392) [17]. In this study, we aimed to prospectively analyze whether lactose or fructose malabsorption is a major cause of CAP by the symptomatic hydrogen breath test (H_2_BT) or diagnostic elimination diet with noticeable improvement at follow-up. 

## 2. Materials and Methods 

### 2.1. Participants and Ethics

From March 2014 until March 2016, a total of 253 eligible patients, aged 7–12 years, presenting with unexplained CAP in five pediatric German gastroenterology centers were investigated to select patients for a randomized controlled group interventional cognitive behavioral training program for functional abdominal pain disorders (FAPD) (NCT02030392) [17]. This study was approved by the institutional review board in Potsdam, Germany and the local ethics committees. According to the study’s eligibility criteria, which were based on Rome III, these patients had suffered from CAP at least once a week for no less than two months [18].

### 2.2. Clinical Evaluation

**Figure 1:**
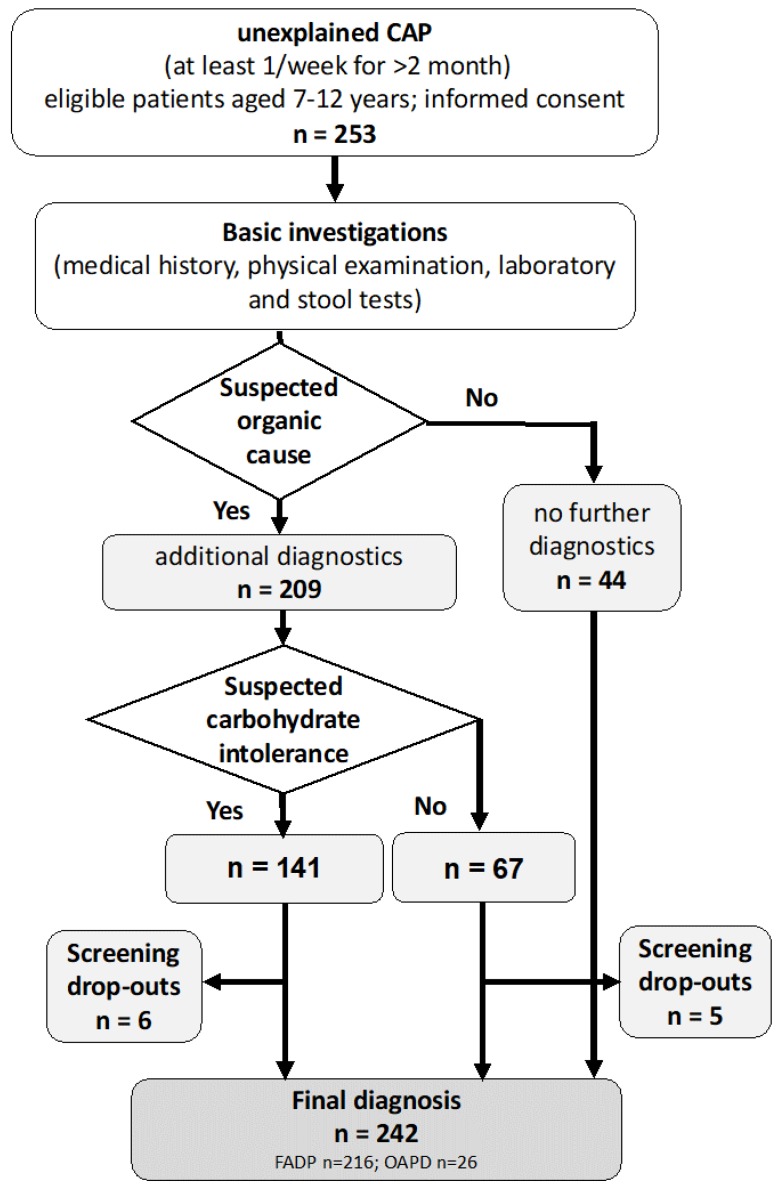
**Diagnostic flow diagram.** A standardized, stepwise medical screening approach was used to classify chronic abdominal pain (CAP) in children. All 253 eligible patients with unexplained CAP underwent basic investigations. Additional diagnostic procedures were initiated in 209 patients depending on each individual’s medical history (e.g., potential alarm features, abdominal pain in relation to dietary intake), and abnormalities identified during the basic investigations (Supplementary Table S1 and Supplementary Figure S1). Carbohydrate intolerance was assumed in 141 patients, if symptoms were reported in relation to fructose or lactose ingestion. Of these, 135 patients were analyzed, six cases were lost to follow-up, or had missing data on the final diagnosis. In total, 242 patients received a diagnosis of an organic disorder or abdominal pain-related functional pain disorder.

Across the study centers, a systematic, stepwise medical screening approach based on current recommendations [19] was followed to identify the organic causes of CAP such as inflammatory bowel disease, celiac disease, or food intolerance, according to the trial protocol [17]. After informed consent, the standardized medical histories of all 253 patients (e.g., pain history, related symptoms, alarm signs like fever, bloody stools, involuntary weight loss, persistent vomiting; see Supplementary Figure S1), thorough physical examination (skin, ear, nose, throat, cardiac, pulmonic, and abdominal examination, body weight, length, blood pressure), and laboratory (complete blood count, erythrocyte sedimentation rate, blood glucose, basal thyroid stimulating hormone, creatinine, urea, gamma glutamyl transferase, glutamate pyruvate transaminase, glutamate oxalate transaminase, alkaline phosphatase, lipase, IgA, C-reactive protein, anti-transglutaminase IgA antibodies, anti-deamidated gliadin IgG antibodies), multistix urine test, and stool tests (including calprotectin, occult blood testing, stool ova, and parasite test) were investigated (Supplementary Tables S1 and S2). Further diagnostic evaluation was initiated depending on the individual clinical picture including alarm features, dietary related symptoms, and abnormalities identified in basic investigations. Additional diagnostic tests such as abdominal ultrasound, endoscopy with biopsies, magnet resonance imaging/computer tomography, upper gastrointestinal x-ray series, fecal pathogen analysis (e.g., Helicobacter pylori antigen), medical treatment attempts, gynecological or psychiatric examination, skin prick test, long-term pH-metry, H_2_ breath test, or an elimination diet were performed to exclude organic causes of CAP such as celiac disease, carbohydrate intolerance, *H. pylori* gastritis, etc. (Figure 1 and Supplementary Table S2). Carbohydrate malabsorption was suspected and further evaluated by the H_2_ breath test, or an elimination diet if gastrointestinal symptoms were reported in association with the consumption of fructose or lactose. The Strengths and Difficulties Questionnaire (SDQ) [20] was applied to the subgroup with functional abdominal pain diagnosis who were eligible for the randomized controlled trial.

### 2.3. Evaluation of Carbohydrate Intolerance

In order to investigate the causal relationship of lactose and fructose intolerance in CAP, we analyzed the presence of symptoms and the abnormal increase during H_2_BT as well as symptom relief during consumption of an elimination diet at the follow-up visit. H_2_BT was performed with 2 g/kg (maximum 50 g) of lactose or 1 g/kg of fructose (maximum 25 g) dissolved in water, which is comparable to quantities used in other studies (Supplementary Tables S3 and S4) [15,21]. After overnight fasting, the hydrogen concentration in exhaled air was measured before, and at 30, 60, 90, and 120 minutes after carbohydrate ingestion [22]. Breath samples were collected after a maximal inspiration followed by a short period of breath holding and prolonged expiration through a mouthpiece [22]. An increase of ≥20 ppm (parts per million) from the basal value was defined as cut-off for a positive H_2_BT result, indicating malabsorption [15]. In addition, a real-time assessment of symptoms occurring during H_2_BT such as abdominal pain, nausea, flatulence, and diarrhea was performed and considered in the evaluation [22]. Furthermore, the parents were instructed to report late-onset symptoms that occurred within the following hours such as diarrhea and abdominal pain. Any typical symptom occurring in a time-related fashion accompanied by an abnormal increase of hydrogen exhalation was regarded as a positive test. Each patient with a positive H_2_BT result and typical symptoms was put on a fructose restricted or lactose free diet. In practice, carbohydrate malabsorption was regarded as unclear in patients with a positive H_2_BT result (ΔH_2_ ≥ 20 ppm) who developed no symptoms. Patients who had undergone previous antibiotic treatment or bowel cleansing received diagnostic elimination diets and no H_2_BT. Diagnostic elimination diets for lactose or fructose were performed for at least seven days. Adequate realization of the carbohydrate restriction diet was ensured by giving detailed instructions to parents to eliminate fructose, lactose, or both in the child’s diet. In addition, they received written information about fructose or lactose intolerance that indicated foods containing fructose and lactose and described the pathology and symptoms. Diagnoses of lactose or fructose intolerance were confirmed by a relief in symptoms after elimination of the respective carbohydrate at follow-up.

### 2.4. Classification of Underlying Causal Disease Groups 

At the end of the diagnostic assessment, the attending pediatric gastroenterologist determined the final diagnosis and leading cause of CAP on the basis of the medical screening (including additional diagnostics) and response to treatment or dietary intervention (including elimination and provocation), which corresponds to other research approaches [8,10]. Functional abdominal pain disorders (FAPD) were diagnosed in children using the Rome III criteria, as Rome IV was only released later on during the study [18,23,24]. Children with carbohydrate malabsorption reporting symptom relief during open-label elimination of the respective carbohydrate at follow-up were classified as intolerant and assigned to the organic abdominal pain disorder (OAPD) group. The persistence of abdominal pain at least once per week during the elimination diet without evidence for organic disease was classified as predominant FAPD. All patients with proven organic gastrointestinal disease were classified as having organic abdominal pain disease (OAPD).

### 2.5. Statistics

Data were analyzed using IBM SPSS Statistics (Version 21) (IBM Deutschland GmbH, Ehningen, Germany) and Microsoft Excel 2013 (Microsoft Corporation, Redmond, WA, USA). On a descriptive level, the arithmetical group means, absolute, and relative frequencies and standard deviations were calculated. Comparisons for categorial variables were analyzed by the chi-square-test (χ^2^) and Fischer’s exact test. For all statistical tests, a significance level of 5% (p-value < 0.05) was used.

## 3. Results

### 3.1. Patient Demographics and History

A total of 242 patients (141 (58%) female), aged seven to twelve years (mean ± SD = 9.71 ± 1.63, median = 10) presented with CAP of unknown origin were analyzed. The majority (71.4%) had suffered from CAP for more than six months, and 39.2% indicated a duration since onset of more than two years (Table 1). Almost two-thirds of the patients (69.2%) had consulted a physician due to CAP more than twice within the last six weeks before the first visit to the study center. Alarm findings were reported in 64 (26%) of the cases (Supplementary Figure S1). In addition, half of the patients reported complete (48%) or partial (56%) absences from school, and disturbed leisure activities (58%) within the last four weeks because of their abdominal pain. In addition, some children suffered from a chronic medical (n = 23, of which the most frequent allergies n = 4, asthma n = 4, diabetes n = 2, obesity n = 2, epilepsy n = 2, atopic dermatitis n = 2) or psychological disorders (n = 6, of which n = 4 attention deficit disorders, n = 1 somatoform disorder and depression, n = 1 emotional disorder and enuresis), according to parent report. Basic diagnostic revealed abnormal results for abdominal examination (n = 43), blood (n = 13), urine (n = 7), and stool (n = 36) (Supplementary Table S1), which led, among other findings, to additional diagnostics in 209 patients (Figure 1 and Supplementary Table S2). Concerning psychiatric symptoms, data from the Strengths and Difficulties Questionnaire parent questionnaire (SDQ) for a subgroup of n = 171 cases with FAPD diagnosis eligible for the RCT are available [20]. Analysis shows a borderline total score in 13.1% and an abnormal total score 16.7%, according to German normative values [25].

The patients’ parents had mostly German citizenship (89% of mothers (mean age 42 ± 5) and 90% of fathers (mean age 44 ± 6)) as sufficient language skills were mandatory for participation in the intervention trial. In addition, their education level was above average (Table 1). About 18% were single parents compared to around 7% of the general population in Germany (Federal Statistical Office 2014) (Table 1). 

### 3.2. Investigation of Carbohydrate Malabsorption

Carbohydrate malabsorption was suspected in 141 out of 253 patients (55.7%) if gastrointestinal symptoms were reported in association with the consumption of fructose or lactose (Figure 1), and analyzed in 135 out 242 (Supplementary Table S2). Accordingly, co-occurrence of lactose and fructose malabsorption was suspected in 109 of the 135 analyzed patients. In order to rule out carbohydrate malabsorption as the cause of CAP, 118 fructose and 114 lactose hydrogen breath tests (H_2_BT) were performed (Table 2). 

H_2_BT revealed an abnormal increase over baseline (ΔH_2_ > 20 ppm) with the presence of typical symptoms in 30% (35/118) of patients after fructose and 18% (20/114) after lactose load. H_2_BT levels did not match the symptoms in six cases of fructose and one of lactose load (Table 2). In the case of H_2_ increase, but lack of symptoms (n = 6 after fructose, n = 1 after lactose breath test), further fructose and lactose diagnostic elimination diets were performed afterward. In addition, in 13 cases with normal H_2_BT (n = 5 fructose H_2_BT and n = 8 lactose H_2_BT), a diagnostic empiric elimination diet was performed in addition to H_2_BT to verify carbohydrate intolerance. H_2_BT was not carried out in 17 patients due to factors influencing H_2_BT reliability such as previous antibiotic treatment or bowel cleansing. Thus, diagnostic elimination diets for both, fructose, and lactose were exclusively performed in these cases.

Complete symptom relief during the diagnostic elimination diet indicated intolerance, and no change represented tolerance. Two patients reported pain reduction during either the fructose or lactose elimination diet; however, CAP persisted at least once per week. Therefore, the elimination diet was regarded as partially beneficial.

Finally, carbohydrate intolerance was suspected in 55 patients: 30 patients after fructose, 17 after lactose, and eight patients after both diagnostic tests (Table 2). These patients and their parents were informed about fructose or lactose intolerance and instructed to eliminate fructose, lactose, or both in the child’s diet. 

### 3.3. Classification of Carbohydrate Malabsorption and Intolerance

From the 55 patients with suspected carbohydrate intolerance, only 10 (18%) reported pain relief after the elimination of the respective carbohydrates in the follow-up (mean time spam for these 10 patients between H_2_BT and final diagnosis: 7.9 (SD 9.9) weeks for fructose and 10.3 (SD 10.7) weeks for lactose; mean time span of diagnostic elimination diet and final diagnosis: 9.3 (SD 2.5) weeks for fructose and 6.5 (SD 9.2) weeks for lactose testing), and carbohydrate intolerance can be considered the exclusive etiological factor for CAP in these cases (Table 3). Thus, abdominal pain persisted in the majority of patients with carbohydrate malabsorption during the elimination of the respective carbohydrates: 82% (14/17) lactose, 87% (26/30) fructose, and 63% (5/8) of combined malabsorption (Table 3). Forty-three of these 45 patients were classified as predominant FAPD with detectable malabsorption after the exclusion of other organic diseases. The two other cases of fructose malabsorption in patients who suffered from a concomitant predominant organic abdominal pain disease (OAPD) fulfilled the diagnostic criteria for celiac disease and gastro-esophageal reflux disease, respectively. Therefore, they were classified as OAPD with detectable malabsorption (Table 3). 

### 3.4. Carbohydrate Malabsorption as an Incidental Finding in Functional Abdominal Pain Disorders versus Intolerance as a Cause of Chronic Abdominal Pain 

Two hundred and forty-two patients with CAP were grouped after basic investigation and additional diagnostics according to their final diagnosis into two groups: children with confirmed organic abdominal pain disorders (OAPD) or children with functional abdominal pain disorders (FAPD). Children with functional abdominal pain disorders (FAPD) made up the largest group of 216 patients (89%). Of these, one-fifth had detectable fructose or lactose malabsorption (n = 43, 20%) (Table 3). According to Rome III criteria, they were classified as having functional abdominal pain (115; 53%), functional abdominal pain syndrome (64; 29%), irritable bowel syndrome (IBS) (23; 11%), functional dyspepsia (4; 2%), or a non-specified condition (12; 6%). An organic cause of CAP was found in 11% (n = 26) of patients. Among them, a distinct portion (n = 10, 38%) suffered from carbohydrate intolerance (Table 3). No statistical differences regarding age, sex, duration of CAP, pain related-disability, school absence, or lactose and fructose intolerance testing were detected among children with OAPD and FAPD (data not shown).

## 4. Discussion

We found a high prevalence of abnormal fructose and lactose hydrogen breath tests among a treatment-seeking patient cohort with unexplained CAP [17]. Interestingly, abdominal pain symptoms still persisted during an elimination diet and after follow-up in more than three-quarters of patients. 

Summing up the results, carbohydrate malabsorption and intolerance was observed in 41% (55 out of 135) of patients tested and 23% (55 out of 242) of patients analyzed, respectively. This represented the most frequent abnormality recorded in the medical screening of our study population. This observation is in line with other studies evaluating children with CAP or irritable bowel syndrome (IBS) that reported even higher rates of malabsorption detected by abnormal breath tests (Supplementary Tables S3 and S4) [8,21,26,27,28,29,30]. Of note, patients in our cohort received diagnostic tests for carbohydrate malabsorption only if they reported abdominal pain associated with dietary intake. This approach might influence the rate of positive H_2_BT observed in our sample. Interestingly, Gijsbers et al. noticed abdominal pain in 25% of children with CAP after lactose load and 48% after fructose load during and after H_2_BT, despite having normal test results [29], suggesting that the test situation itself may trigger symptoms due to an expectation bias, having fasted or being stressed. In contrast, we rarely observed symptoms in H_2_BT negative patients in our cohort, which may be due to the lower fructose load of 1 g/kg compared with 2 g/kg body weight used in the study of Gijsbers [29]. 

Indeed, methodological aspects of H_2_BT in children may influence the detection rate of carbohydrate malabsorption by H_2_BT [31]. We administered a solution of 2 g of lactose per kilogram body weight up to a maximum of 50 g, which is within the upper dose range of 0.5 to 2 g/kg up to an absolute amount of 25 or 50 g used in pediatric studies (Supplementary Table S3) [11,22,27,29,30,31,32]. In the absence of a gold standard for fructose H_2_BT, we administered a fructose solution of 1 g per kilogram body weight up to a maximum of 25 g, which is within the dose range of 0.5 to 2 g/kg up to a maximum of 10 to 50 g used in recent pediatric studies (Supplementary Table S4) [9,11,21,29,33,34,35]. Of note, increasing the fructose load up to 1.4 g/kg body weight on average resulted in increases in hydrogen production and H_2_BT positivity [9]. Furthermore, the capacity to absorb fructose increases with age, and should be considered when interpreting H_2_BT results and responses to fructose-restricted diets in the first decade of life [33]. In our cohort, which included patients aged 7 to 12 years, the capacity to absorb fructose seems to be already sufficient, as this did not lead to a higher detection ratio of fructose malabsorption by H_2_BT compared to other study samples using the same fructose load (Supplementary Table S4) [10,28,34]. In the absence of an unequivocal reference test for lactose or fructose malabsorption, the use of H_2_BT is still recommended to identify children with suspected carbohydrate malabsorption that causes symptoms [11,22]. Furthermore, lactose breath testing has been shown to be more reliable than medical history or self-reporting of lactose intolerance [15,36]. The positive predictive value of self-reported lactose intolerance is very low according to studies in adult patients [36]. It is assumed that this might also be in children [15]. However, pediatric studies are missing.

Although carbohydrate malabsorption is frequently detected in children, which corresponds to our findings, symptom relief has not been achieved by elimination of the respective carbohydrate in several randomized controlled trials [29,37]. We report lower dietary response rates to lactose-free or fructose-restricted diet (24% and 18%, respectively) than other studies [8,10,21,28,30,35,38]. However, the best dietary response rates (81–95%) have been reported in patients diagnosed with lactose or fructose intolerance [9,30,38]. In our study, dietary response was defined as relief in abdominal pain, whereas other studies rated abdominal pain reduction as a response [8,35]. In addition, several studies assessed the dietary response after one or two months [10,35]. Thus, a later assessment of dietary response may also influence the response rate as a decrease in the impact of diet has been reported [30]. In fact, carbohydrate malabsorption was not the primary cause of CAP in our and several other studies [29,32]. In a study of 220 children, Gijsbers et al. found no decisive relationship between CAP and lactose or fructose malabsorption [29]. In their study, children with positive H_2_BT (ΔH_2_ ≥ 30 ppm) results received an elimination diet, and in the case of persisting CAP, an open provocation test. Thereafter, a double-blinded placebo-controlled (DBPC) provocation test was performed in H_2_BT positive children who reacted to the open provocation test. Interestingly, none of these children received positive results in the DBPC provocation test, so the existence of lactose or fructose intolerance was excluded [29]. Possibly, the lactose and fructose dosages used were too low to induce symptoms, as the doses were administered throughout the day. Thus, a standardized, reliable DBPC provocation test for the pediatric population is still missing. Genetic screening for adult type hypolactasia correlates best with duodenal lactase activity in children over 12 years of age and is recommended as a first stage screening test for adult type hypolactasia [39]. However, even children with biopsy-proven decreased intestinal lactase activity displayed lactose tolerance toward dietary lactose intake without developing gastrointestinal symptoms [7]. Interestingly, the recovery rate from CAP in the study by Lebenthal et al. was independent of a lactose elimination diet in both lactose absorbers and non-absorbers [7]. 

A decrease in gastrointestinal symptoms, particularly abdominal pain, has been reported after dietary-restricted fructose intake among both fructose breath test positive [9,10,11,28,34] and negative patients [37]. So far, only two randomized controlled trials evaluating nutritional effects on CAP in children have been conducted [37,40]. Dietary fructose restriction led to an improvement in CAP and secondary symptoms independent of the H_2_BT result [37], and a diet low in fermentable oligo-, di-, mono-saccharides, and polyols (FODMAPs) for 48 h decreased abdominal pain frequency in children with IBS [40]. The study done by Wirth et al. might especially reflect the high placebo effect of dietary restrictions in patients with abdominal pain [37] and may explain the absent response toward DBPC carbohydrate provocation in H_2_BT positive patients [29]. Currently, insufficient evidence exists to recommend dietary restrictions involving lactose or fructose elimination or a long-term FODMAP diet, which may lead to nutritional deficits in children with CAP in general [14,21,41,42]. 

Carbohydrate malabsorption was frequently associated with FAPD in our cohort whereas a higher occurrence of lactose malabsorption, of which 37% of cases were detected by H_2_BT (cut off ≥ 20 ppm), has already been reported in children with both organic and functional gastrointestinal diseases [43]. The highest symptomatic lactose intolerance ratio was found among children with irritable bowel syndrome (47.8%) in that study [43]. Moreover, adult IBS patients with predominant abdominal pain benefit from fructose-restricted diets measured by a visual analogue scale independent from the results of fructose H_2_BT [44]. According to the authors, this indicates visceral hypersensitivity rather than intolerance [44]. Indeed, we also identified a few patients who reported a pain reduction during an elimination diet of fructose and lactose. A significant reduction in abdominal pain intensity and frequency was reported during a fructose restricted diet in children with CAP [10]. However, we did not quantify the effect of dietary restriction in our study by a shared standardized pain-scale or by a diary to assess abdominal pain or symptom relief during the elimination diet. Apart from detailed dietary instructions and written information, no further measures to increase dietary compliance were performed in our study (e.g., control of food intake by a nutritional journal). The underlying pathophysiology of FGID with predominant abdominal pain in children is poorly understood and influenced by several factors such as child maltreatment, stressful events, visceral hyperalgesia, lower gastric motility, and alteration in the microbiota [45,46]. The impacts of these factors cannot be excluded and need to be taken into account in the interpretation of our results from a treatment seeking cohort. Further prospective randomized controlled studies among children are needed to evaluate the role of carbohydrate intake and long-term effects on the gut microbiome composition and function in abdominal pain predominant FGID [14]. 

Furthermore, other types of food intolerance including non-celiac gluten or wheat sensitivity, histamine intolerance, polyols (such as sorbitol), sucrose, and starch should be considered in children with CAP [12]. We also diagnosed one patient with sorbitol intolerance in our cohort. In addition to the clinical picture, an appropriate dietary and nutritional assessment is necessary to identify patients suspected of having food intolerances to specific components and initiate a short-term removal followed by a re-introduction to assess symptom response [12].

Making the diagnosis of FAPD or carbohydrate intolerance can be a challenge, as reliable biomarkers are missing and diagnostic tests have their limitations; therefore, a structured diagnostic algorithm for CAP is mandatory [13,19,43,47,48]. In order to select patients with FAPD for an interventional trial, we established a detailed diagnostic program based on current evidence, guidelines, and clinical practice. In our study, the majority of abnormal H_2_BT results (76% for lactose and 82% for fructose) had no clinical relevance, as CAP persisted after elimination of the respective carbohydrate. Indeed, H_2_BT is not sufficient to ensure and verify carbohydrate intolerance in children with CAP [21,43,49]. Thus, the diagnosis of fructose or lactose intolerance should be verified by symptoms during H_2_BT, elimination diet, open provocation, and further diagnostic workup, respectively [21,29,43]. Thereby, carbohydrate malabsorption diagnosed by an abnormal H_2_BT could be separated from clinically significant intolerance or visceral hypersensitivity hampering everyday life. Diarrhea and flatulence are regarded as the most specific symptoms of lactose intolerance in H2BT positive patients [32]. A careful assessment of symptoms after fructose ingestion using a validated questionnaire may identify symptomatic patients with intestinal fructose intolerance rather than performing H_2_BT with low specificity to diagnose fructose malabsorption [21]. Furthermore, fructose or lactose intolerance may be secondary to other gastrointestinal diseases affecting digestion and the absorption of nutrients in some cases (e.g., celiac disease, food allergy, and inflammatory bowel disease) [13]. For these patients, dietary restriction of lactose or fructose is not a sufficient treatment of the underlying disorder, and the primary cause of carbohydrate intolerance and abdominal pain needs to be clarified. Moreover, growing children and adolescents should be protected from low calcium, vitamin D, or C diets. In addition, carbohydrate malabsorption as a trigger factor for FAPD should be mentioned in the context of an informative conversation on disease models of FAPD or in cognitive-behavioral pain management programs for children [50]. 

The results of the study need to be discussed in terms of their strengths and limitations. A systematic medical screening including alarm findings, physical examination, laboratory, urine, and stool tests was performed in all children presented with CAP of unknown origin in a defined age group to identify an organic cause of CAP. Accordingly, additional diagnostics and treatments were carried out to confirm the diagnosis. However, diagnostic tests of fructose and lactose intolerance were only performed in individuals reporting dietary related symptoms regarding fructose and lactose consumption. Furthermore, we did not systematically screen for other types of food intolerance as a cause of CAP. In addition, the beneficial effect of dietary restriction may be underestimated in our study as we did not quantify the reduction in pain intensity or frequency and amelioration of symptoms during elimination of the respective carbohydrate. The data for this report were derived from a stepwise medical screening of children presenting with chronic abdominal pain of unknown origin in order to identify individuals suffering from FAPD eligible for an interventional RCT. Therefore, only cross-sectional screening data are available for this report and long-term data are missing. Future studies with longitudinal designs, closely monitored dietary adherence, and quantification of intensity and frequency of symptoms are needed to gain further insight into the role of carbohydrate malabsorption in FAPD.

## 5. Conclusions

We frequently found abnormal fructose and lactose breath tests in children with CAP and thus question the usefulness of hydrogen breath testing to detect carbohydrate intolerance as abdominal pain still persisted despite following a lactose- or fructose-restricted diet in the majority of patients at follow-up. Carbohydrate malabsorption in these patients was an incidental finding rather than the cause of functional abdominal pain and requires the implementation of appropriate standardized diagnostic testing and therapy to avoid unnecessary dietary restrictions in children suffering from FADP. The role of carbohydrate malabsorption in the pathogenesis of FAPD still remains a matter of discussion and needs further investigation.

## Figures and Tables

**Table 1:** Patient demographics and history.

**Patients (n = 242)**	Girls 141 (58.3%)		Boys 101 (41.7%)	
**Age (years)**	mean 9.71 (SD ± 1.63) median 10 range 7–12			
**Weight (kg)**	mean 36.07 (SD ± 12.37) range 19.5–75.6			
**Length (cm)**	mean 140.78 (SD ± 12.27) range 114.7–170.0			
**Duration of abdominal pain** (n = 221)	2–6 months 61 (28.6%)	6–24 months 69 (32.2%)		>24 months 91 (39.2%)
**Pain-related** (n = 219)	partial school absence 55.7%	disturbed family life 26.9%		disturbed leisure activities 57.6%
**Consultation of physician due to abdominal pain in the last 6 weeks** (n = 227)	Never 8 (3.5%)	1–2 times 62 (27.3%)	Times 105 (46.3%)	>6 times 52 (22.9%)
	Mothers		Fathers	
**Age**	mean 42 ± 5 years		mean 44 ± 6 years	
**German Citizenship**	89%		90%	
**Graduation**	47% A level/Advanced Vocational Certificate of Education 39% O-level		53% A level/Advanced Vocational Certificate of Education 25% O-level	
**Relationship status of parents**	82% living together 18% single parent			

**Table 2:** Diagnostic tests for carbohydrate malabsorption in 135 children.

Diagnostic Test	Total Number of Cases	Results of Diagnostics		
**H_2_BT**		**H_2_ < 20 ppm** **No Symptoms**	**H_2_ ≥ 20 ppm** **No Symptoms**	**H_2_ ≥ 20 ppm** **With Symptoms**
**Fructose**	118	77	6	35
**Lactose**	114	93	1	20
**Diet**		**Pain-Persistence**	**Partial Pain-Reduction**	**Complete Pain-Relief**
**Fructose-Restricted**	28	12	2	14
**Lactose-Free**	26	13	2	11

**Table 3:** Classification of carbohydrate malabsorption and intolerance after H_2_BT and the elimination diet at follow-up.

Carbohydrate	Intolerance	Malabsorption		N
	OAPD	OAPD	OAPD	
**Fructose**	4	2	24	30
**Lactose**	3	0	14	17
**Lactose + Fructose**	3	0	5	8
**N**	10	2	43	55
		45		

OAPD = organic abdominal pain disorder; FAPD = functional abdominal pain disorder.

## Supplementary Materials

The following are available online at https://www.mdpi.com/2072-6643/11/12/3063/s1. Supplementary Table S1: Basic diagnostic findings in children with chronic abdominal pain; Supplementary Table S2: Additional diagnostic examinations in 209 children with chronic abdominal pain; Supplementary Table S3: Prevalence of abnormal lactose H_2_BT and response to elimination diets in various studies investigating CAP in children; Supplementary Table S4: Prevalence of abnormal fructose H_2_BT and response to elimination diets in various studies investigating gastrointestinal disease in children; Supplementary Figure S1: Alarm findings (n = 87) in 64 out of 242 children with chronic abdominal pain.
