# Supplementary material for: Roles of Lactose and Fructose Malabsorption and Dietary Outcomes in Children Presenting with Chronic Abdominal Pain
Source: Nutrients. 2019 Dec 16;11(12):3063. doi: 10.3390/nu11123063 (PMC6950325; doi:10.3390/nu11123063)
Supplement: Supplementary file 1 [file nutrients-11-03063-s001.pdf]

Supplemental Table S1: Basic diagnostic findings in children with chronic abdominal pain (numbers indicate n (%)).

	alarm findings	abdominal examination	blood	urine	stool
**abnormal**	**64** (26%)	**43** (17%)	**31** (13%)	**7** (3%)	**36** (15%)
**normal**	**177** (73%)	**198** (82%)	**199** (82%)	**164** (68%)	**183** (76%)
**missing information**	**1** (1%)	**1** (1%)	**12** (5%)	**71** (29%)	**23** (9%)
**total**	**242**	**242**	**242**	**242**	**242**

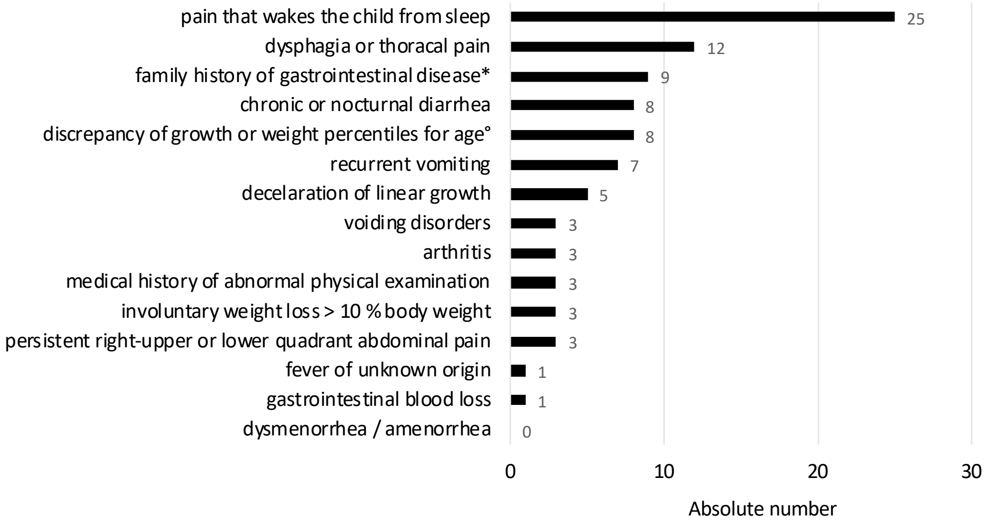

Supplemental Figure S1: Alarm findings (n=87) in 64 out of 242 children with chronic abdominal pain. *e.g. chronic inflammatory disease/ celiac disease/ peptic ulcer or other gastrointestinal diseases. ° according to Kromeyer and Hausschild growth charts. Summing up the number of red flags per patient, at least one red flag was reported resp. observed in n=42 cases, two red flags in n=19; three red flags in n=1, and four red flags in n=2 cases.

Supplemental Table 2: Additional diagnostic examinations in 209 children with chronic abdominal pain.

	Abdominal ultrasound	endoscopy	carbohydrate intolerance tests	others
**examined patients**	**128**	**46**	**135**	**10**
**abnormal results**	**12** (9%)	**12** (46%)	**55** (41%)	**4** (40%)

Others include MRI, CT, bowel contrast study, sorbite breath test, fecal helicobacter pylori antigen, prick skin test, and gynecological examination.

Supplemental Table S3: Prevalence of abnormal lactose H_2BT and response to elimination diets in various studies investigating CAP in children.

authors	no. of patients	Age (y) mean±SD [range]	dosage	cut-off	Detection rate %	Dietary response (n) %
Ceriani 1988	32	8.13 ±2.46 #	2g/kg max.50 g	#	75%	58.3%
Winter 1990	62	# [2-16]	2g/kg max.50 g	Δ≥25ppm	28.3%	(16/17 LI) 94.1%
Ockeloen 2012	91	8.4 [1.8-17.8]	2g/kg	≥20ppm	41%	(17/18 LI) 95.4%
Gijsberg 2012	210	8.8 [4.1-16]	2g/kg max.50 g	Δ≥30ppm	27%	(24/38) 63%
Garg 2014	62	# [4-18]	2g/kg max.50 g	Δ≥20ppm	58%	(20/34) 58.8%

Glatstein<br>2018	95	10.6 ±3.15<br>[6-18]	2g/kg max.50 g	Δ≥20ppm	66.3%	(40/95)<br>38%
Present study	114	9.86 ±1.60<br>[7-12]	2g/kg max.50 g	Δ≥20ppm	17.5%	(6/25)<br>24%

# = no data available; LI= lactose intolerant; SD = standard deviation; Δ = value measured – baseline value.

Supplemental Table S4: Prevalence of abnormal fructose H_2BT and response to elimination diets in various studies investigating gastrointestinal disease in children.

**authors**	**no. of patients**	**Age (y) mean±SD [range]**	**disease**	**dosage**	**cut-off**	**Detection rate %**	**Dietary response (n) %**
Gomara<br>2008	32	12.9[1]<br>[7-17]	CAP	1, 15 or 45 g<br>absolute	Δ≥20ppm	0/9 (1g) 0%<br>3/10 (15g)<br>30%<br>8/13 (45g)<br>61%	(9/11 FI)<br>81.8%
Tsampalieros<br>2008	89	#<br>[3.7-18.7]	Not<br>defined	1g/kg<br>max. 25-50 g	Δ≥20ppm	55/89<br>62%<br>29/89*<br>33%*	
Jones<br>2011	760	#<br>[0-15]	Not<br>defined	0.5g/kg max.10<br>g	Δ≥10ppm	1-5y 66.6%<br>6-10y 40.4%<br>11-15y<br>27.1%	n.d.
Gijsberg<br>2012	210	8.8<br>[4.1-16]	CAP	2g/kg max.50 g	Δ≥30ppm	57/210*<br>27%*	(24/38)<br>63%
Wintermeyer<br>2012	278	8.4<br>[3-14]	CAP	1g/kg<br>max.25 g	Δ≥20ppm	117/278<br>42%	58.7%
Escobar<br>2014	222	10.5<br>[2-19]	CAP	1g/kg<br>max.25 g	Δ≥20ppm	121/222<br>55%	(93/112) 76.9%
Wirth<br>2014	103	Median<br>8.8<br>[3.4-16.4]	CAP	1g/kg<br>max.25 g	Δ≥20ppm	55/103<br>53%<br>33/103*<br>32%*	
Hammer<br>2018	82	10.7 ±3.5<br>[5-17.6]	Not<br>defined	1g/kg<br>max.25 g	-	33/82<br>40%	(15/33)<br>45%
Martinez-Azcona<br>2019	71	9.5±2.8	CAP	1g/kg<br>max.25 g	Δ≥20ppm	29/71<br>40.8%	21/29<br>73.4%
Present study	118	9.81±1.58<br>[7-12]	CAP	1g/kg max.25 g	Δ ≥20ppm	35/118*<br>30%*	(7/38)<br>18%

# = no data available; * abnormal hydrogen breath test with symptoms; CAP = chronic abdominal pain; FI = fructose intolerance; Δ = value measured – baseline value; [1)] mean weight 55.2 kg.
